# Normal tissue complication probability modeling for late rectal bleeding after conventional or hypofractionated radiotherapy for prostate cancer

**DOI:** 10.1016/j.ctro.2024.100886

**Published:** 2024-11-10

**Authors:** Christian A.M. Jongen, Ben J.M. Heijmen, Wilco Schillemans, Andras Zolnay, Marnix G. Witte, Floris J. Pos, Ben Vanneste, Ludwig J. Dubois, David van Klaveren, Luca Incrocci, Wilma D. Heemsbergen

**Affiliations:** a Department of Radiotherapy, Erasmus MC Cancer Institute, University Medical Center Rotterdam, Dr. Molewaterplein 40, 3015 GD Rotterdam, the Netherlands; b Department of Radiation Oncology, The Netherlands Cancer Institute-Antoni van Leeuwenhoek, Plesmanlaan 121,1066 CX Amsterdam, the Netherlands; c Department of Radiotherapy-Oncology, Ghent University Hospital, Corneel Heymanslaan 10, 9000 Ghent, Belgium; d Department of Radiation Oncology (MAASTRO), GROW – Research School for Oncology and Reproduction, Maastricht University Medical Center, Dr. Tanslaan 12, 6229 ET Maastricht, the Netherlands; e Department of Precision Medicine, Grow – Research School for Oncology and Reproduction, Maastricht University Medical Center, Dr. Tanslaan 12, 6229 ET, Maastricht, the Netherlands; f Department of Public Health, Erasmus MC, University Medical Center Rotterdam, Dr. Molewaterplein 40, 3015 GD Rotterdam, the Netherlands

**Keywords:** Prostate cancer, Radiotherapy, Hypofractionation, Rectal Bleeding, Normal tissue complication probability

## Abstract

**Purpose:**

To develop a single NTCP model for grade ≥ 2 late rectal bleeding (G2 LRB) after conventional or hypofractionated radiotherapy for prostate cancer.

**Methods and Materials:**

The development dataset consisted of prostate cancer patients (n = 656) previously randomized to conventional (39 x 2 Gy) or hypofractionated (19 x 3.4 Gy) external beam radiotherapy with N = 89 G2 LRB cases. Candidate predictors were obtained from literature. We fitted five separate logistic regression models to the data, each with one of the following dose parameters as candidate predictors in biological effective dose (BED), assuming α/β = 3 Gy: Equivalent uniform dose (EUD) with n = 0.1, EUD with n = 0.2, the relative volume receiving ≥ 111.9 Gy in BED (V111.9, the equivalent of physical V70 for a conventional schedule), minimum BED to the hottest 0.1 cm^3^ (D_0.1cm3_) or 2 cm^3^ (D_2cm3_). Previous abdominal surgery was included in every model and fractionation schedule was tested as predictor in each model. A sensitivity analysis was performed by varying the α/β-ratio, n and dose-volume cutoff.

**Results:**

The pre-selected candidate dosimetric predictor and previous abdominal surgery were significantly associated with the outcome in all five models. Fractionation schedule was eliminated by the backward scheme in only the EUD (n = 0.1), D_0.1cm3_ and D_2cm3_-based models. In internal validation these models showed AUC’s of 0.64, 0.60 & 0.62, respectively. The sensitivity analyses showed that EUD models with n ≥ 0.15 and / or α/β ≥ 4 Gy failed, and EUD models based on α/β = 2 Gy with n = 0.05–0.2 showed good fits as well.

**Conclusions:**

Our trial data set with different fractionation schedules offered the unique possibility to generate unbiased BED-based models. EUD (n = 0.1), D_0.1cm3_ and D_2cm3_ performed overall best in predicting G2 LRB; with α/β = 2 Gy equally good models were obtained. External validation is required to confirm our results.

## Highlights

• We fitted NTCP models for rectal bleeding using data from a randomized trial with hypo- & standard fractionation.
• Biological Effective Dose assuming α/β = 2 Gy appeared to fit better than α/β = 3 Gy.
• EUD (n = 0.1), D_0.1cm3_ and D_2cm3_-based models had superior model characteristics.

## Introduction

Late rectal bleeding (LRB) is a frequently reported symptom of gastrointestinal toxicity after external beam radiotherapy (EBRT) for prostate cancer. Several normal tissue complication probability (NTCP) models were developed describing the relation between the probability of LRB and dosimetric and clinical parameters [1], [2]. These models were typically fitted on data from conventional schedules with fraction sizes between 1.8 and 2 Gy and mainly describe risks as a function of physical dose. However, hypofractionated radiotherapy has recently become the new clinical standard for EBRT for prostate cancer since several large clinical trials demonstrated equivalence to conventional EBRT schemes with respect to tumor control and late toxicity [3], [4], [5], [6]. Therefore, there is an increasing need for NTCP models based on dosimetric parameters that are adjusted in accordance with the linear-quadratic model [7], such that they are valid for multiple fractionation schedules. Moreover, using a dataset with various fractionation schedules, offers the opportunity to estimate α/β and volume effects.

Commonly used dosimetric parameters in NTCP models for LRB include the equivalent uniform dose (EUD) [8], [9], [10], [11], [12], [13] and the relative volume receiving a certain dose D or more (V_D_) [10], [14], [15], [16], [17], [18], [19], [20], [21]. Another widely applied dosimetric parameter is the minimum dose in the most exposed X cm^3^ (D_xcm3_) which is used in predictive models for rectal toxicity after EBRT and brachytherapy (BT) for cervical cancer [22], [23].

The objective of the current study is to fit models for grade ≥2 LRB based on dosimetric parameters expressed in biological effective dose (BED). The dataset that is used contains dosimetric and clinical parameters from patients who were treated with either a conventional (CF) or moderate hypofractionated (HF) schedule in the Dutch multicenter randomized HYPRO trial [24], [25], [26]. With this study design we are able to evaluate which BED based model best describes the relationship between dose and LRB for both trial arms.

## Methods and materials

### Study population

We used the dataset of the HYPRO trial, in which 820 intermediate- to high-risk prostate cancer patients were recruited between 2007 and 2010 and randomized between 78 Gy in 39 fractions of 2 Gy (CF) or 64.6 Gy in 19 fractions of 3.4 Gy (HF) [24], [25], [26]. Patients in the CF group were given 5 fractions a week, resulting in an overall treatment time of about 8 weeks. Patients in the HF groups were given three fractions a week for an overall treatment time of about 6.5 weeks [24], [25], [26]. The trial was approved by the medical ethics committee of the Erasmus Medical Centre in Rotterdam, The Netherlands (06–045). All patients provided written informed consent. A total of 782 patients were previously analyzed for the late toxicity endpoint analysis [27]. From this group we selected patients with at least 12 months follow-up without clinical recurrence and with at least one follow-up visit after 12 months with toxicity evaluation (N=726). In a next step we excluded patients for whom no 3D planning data were available (N=55) and we excluded data from one deviating center who only provided dose data for one arm (N=15), leaving 656 patients for the current analysis. Patient characteristics are reported in Supplementary Material Table S1.

### Treatment and follow-up

The six centres, whose patient data were included, all used image guided (IG) intensity modulated radiotherapy (IMRT). The study protocol dictated that less than 60% of the total rectal volume or less than 50% of the rectal wall volume should receive 83% of the prescribed dose. Additionally, the mean anal canal dose was constrained to 78% of the prescribed dose, or to 74% if the anal wall was delineated [24]. The rectum including the anal canal was delineated on the planning CT. Depending on seminal vesicle involvement risk, these were treated with the prescribed dose, reduced dose or no dose. Planning target volume (PTV) margins between 3 and 10 mm were applied, depending on the centre, but equal for both arms in each centre. Other important differences in local treatment protocol were the application of MRI-based prostate delineation in one centre and the use of an endorectal balloon for rectal wall sparing in another centre [28], [29]. Further details can be found elsewhere [24], [25], [26], [28]. Patients were asked to complete self-assessment questionnaires 6, 12, 24, 36, 48 and 60 months after the start of radiotherapy, as reported elsewhere [24].

### Endpoint definition and scoring

The current endpoint of interest is grade ≥2 late rectal bleeding (G2 LRB) within 5 years after radiotherapy. According to the criteria set by the Radiation Therapy Oncology Group (RTOG) and European Organization for Research and Treatment of Cancer (EORTC), intermittent rectal bleeding should be scored as grade 2 late radiation morbidity [30]. This is in line with Common Terminology Criteria for Adverse Events (CTCAE v5) which defines G2 LRB in case of moderate to severe symptoms and/or a treatment for bleeding is indicated [31]. Therefore we scored G2 LRB if the case report forms on late toxicity (scheduled every 6 months) indicated any form of medication or medical intervention such as hyperbaric oxygen treatment, transfusion, or laser coagulation treatment. To improve the dataset, available patient-reported symptom questionnaires were checked for the question “*did you experience blood in your stools in the past week: no, a little, quite a bit, very much*”, which is identical to the question asked in the EORTC PR25 module (which has a recall period of 4 weeks) [32]. We labeled G2 LRB in case of moderate bleeding on ≥2 questionnaires or severe bleeding on ≥1 questionnaire. For patients with a clinical recurrence, we excluded rectal bleeding events after this time point.

### Dose calculations

The rectal contours were outlined from the anus (at the ischial tuberosities) to the bottom of the sacro-iliac joints. Prior to establishment of dosimetric features, planned doses distributions of the rectum were retrieved and converted to BED voxelwise. We applied an α/β ratio of 3 Gy, since this is the value that is broadly assumed for late rectal toxicity [33], [34], [35], [36]. Fig. S1 shows BED’s and corresponding physical dose in 19 or 39 fractions. We assumed that the difference in overall treatment time between the fractionation schedules did not impact rectal toxicity.

### NTCP model development

Instead of starting with a large set of dosimetric candidate predictors and attempt to select the most predictive ones, we performed a priori selection of candidate dose parameters based on a literature study. This approach was taken to fulfil the 10 events-per-variable (EPV) requirement [37].

Five separate models (models A to E) were fitted, each with one of the following dose parameters as candidate predictor: V111.9, EUD(n=0.1), EUD(n=0.2), D_0.1cm3_ and D_2cm3._ This was based on literature, as described in the following paragraph.

Medium-to-high doses of between 50 and 75 Gy are reported to be predictive for LRB in CF, with V60 and V70 in physical dose as the most frequently reported NTCP parameters [10], [14], [15], [16], [17], [18], [19], [20], [21]. 70 Gy in physical dose for a conventional schedule (39x2 Gy) converts to 111.9 Gy in BED, assuming an α/β ratio of 3 Gy. Therefore V111.9 was chosen as candidate dose predictor.

Selection of EUD n values of 0.1 and 0.2 was based on the range of best fit value between 0.06 and 0.24 reported in Fiorino’s review [1] and a recent validation study [38]. D_0.1cm3_ and D_2cm3_ were chosen since they were found to be predictive for rectal toxicity [23] and LRB [22] after EBRT and BT for cervical cancer. From literature we also obtained ‘previous abdominal surgery’ (ABD SURG) and ‘use of anticoagulation / cardiovascular history’ (not available in our dataset) as relevant clinical parameters [9], [11], [13], [18], [20]. Whether or not the patient was treated with hypofractionated radiotherapy was also added as a candidate predictor which was named HYPOTREAT. In case HYPOTREAT was a significant factor in a final model, it was concluded that the applied BED-based dosimetric parameter with ABD SURG did not describe the dose–response independently from fractionation schedule.

The models were fitted through multivariable binary logistic regression with backward elimination. Akaike Information criterion (AIC) was used as backward selection criterion. To reduce overfitting, regression coefficients were shrunk with a bootstrap-based uniform shrinkage factor, after which the intercept was re-estimated [39], [40]. The shrinkage factor was estimated based on 300 bootstrap samples. The Odds-Ratio (OR) is reported for all predictors in the final models. Apparent model performance was evaluated by the Integrated Calibration Index (ICI) [41], calibration slope and calibration intercept as measures for calibration. The ICI was calculated by taking the mean of the absolute differences between a loess-based calibration curve and the line of perfect calibration for the predicted probabilities [41]. The lower the ICI value, the better the calibration. The Area under the ROC curve (AUC) was evaluated for discriminative power and the Brier score for predictive power. For internal validation, performance measures were corrected for optimism with a bootstrapping procedure with 1000 samples. <0.1% of the bootstrap samples resulted in models with only an intercept. These were excluded from the calculation for optimism. Analyses were performed in MATLAB (The MathWorks Inc. Natick, Ma).

### Sensitivity analysis

We performed sensitivity analysis to test the effect of α/β ratio assumption and the selected dose parameters. Details are described in Section 3 of Supplementary Materials.

## Results

A total of 89 of the 656 patients (14%, 33 in CF & 56 in HF) included in this study were scored as G2 LRB; n=77 scores based on case report forms and an additional n=12 (n=7 HF) based on the questionnaires. Two bleeders were not scored as events since severe hemorrhoids were reported to be the cause. Corresponding cumulative incidences at 5 years were 10.7% for CF vs 18.2% for HF (p=0.007). Eighty LRB events (90%) occurred within 3.5 years. Follow-up was adequate with n=557/656 (85%) having LRB endpoint or >3.5 year follow-up, and only n=7 patients had follow-up <2 year.

### NTCP models

**Fig. 1:**
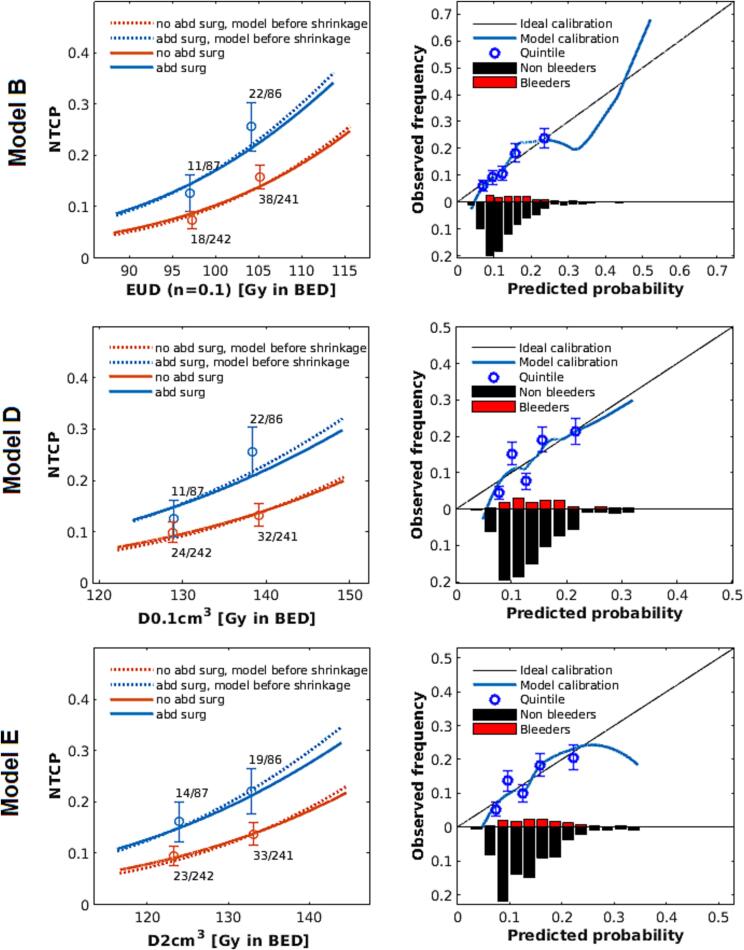
The models based on EUD (n = 0.1), D_0.1cm3_ and D_2cm3_ (B, D and E). The dashed line shows the model before model coefficients were shrunk. The calibration plots show the apparent calibration based on quintiles and loess curve for the unshrunk models. The error bars show standard deviations. The histogram shows the distribution of predicted probabilities for bleeders and non-bleeders. Probabilities of outliers were not included in the model plot, but were in the calibration plots.

Expressions of the final models are shown in Table S2. HYPOTREAT was not eliminated in the models based on V111.9 and EUD(n=0.2) (models A and C, Fig. S2, Table 1). Since the applied BED-based dosimetric parameters in these models did not describe the dose–response independently from dose fractionation schedule, we did not further consider these models. The three models based on EUD(n=0.1), D_0.1cm3_ and D_2cm3_ (models B, D & E, Fig. 1, Table 1) all predict probabilities roughly ranging between 0.05 for relatively low dose levels to around 0.4 for the category of patients with higher dose levels in the rectum. The applied shrinkage factors to these models were 0.93, 0.90 and 0.88, respectively. Associations between the dosimetric predictors and the outcome were similar across the three models (Table 1).

**Table 1:** Predictors of the fitted models with OR and p-value and the OR in the final models.

**Fitted models before shrinkage**					**Final models after shrinkage**
**Model**	**Predictors**	**OR (95% CI)**		**p-value**	OR
**A**	V111.9 in BED HYPOTREAT ABD SURG	1.06 (1.02 – 1.10)* 1.77 (1.10 – 2.83) 1.89 (1.17 – 3.05)		0.004 0.017 0.009	1.05* 1.67 1.77
**B**	EUD(n = 0.1) in BED ABD SURG	1.08 (1.04 – 1.12) 1.88 (1.16 – 3.03)		<0.001 0.010	1.07 1.80
**C**	EUD(n = 0.2) in BED HYPOTREAT ABD SURG	1.05 (1.02 – 1.08) 1.66 (1.03 – 2.67) 1.92 (1.19 – 3.11)		0.003 0.038 0.008	1.04 1.58 1.81
**D**	D_0.1cm3_ in BED ABD SURG	1.05 (1.02–1.09) 1.82 (1.13 – 2.93)		0.004 0.013	1.05 1.72
**E**	D_2cm3_ in BED ABD SURG	1.06 (1.02 – 1.09) 1.83 (1.14 – 2.95)		0.001 0.013	1.05 1.70

*OR per 1% increase in volume.

### Model performance

The apparent loess curve based calibration plot of the EUD (n=0.1)-model (Fig. 1) shows a general underestimation of predicted probabilities in an area between 0.1 and 0.2. Predicted probabilities in the area between 0.3 and 0.4, which consisted of few patients, were generally overestimated. Compared to the EUD(n=0.1)-model, the quintiles in the calibration plots of the D_0.1cm3_ and D_2cm3_-models appear to lie further from the line of perfect calibration.

Table 2 contains the apparent performance measures for the models before shrinkage and the corrected performance for the shrunk final models.

**Table 2:** Apparent performance measures for the models before shrinkage and corrected performance measures for the final models in which fractionation schedule was eliminated.

Measure of performance	**Apparent** Model before shrinkage	**Corrected** (95% CI) Final model, after shrinkage
**Model B (EUD (n=0.1))**		
AUC	0.65	0.64 (0.58–0.69)
ICI	0.022	0.019 (0–0.037)
Calibration intercept	0.00	0.02 (−0.54 – 0.88)
Calibration slope	1.00	1.01 (0.68 – 1.49)
Brier score	0.1139	0.1151 (0.0964 – 0.1325)
**Model D (D_0.1cm3_)**		
AUC	0.62	0.60 (0.55 – 0.65)
ICI	0.008	0.007 (0 – 0.027)
Calibration intercept	0.00	0.02 (−0.56 – 1.19)
Calibration slope	1.00	1.01 (0.68 – 1.65)
Brier score	0.1147	0.1160 (0.0983–0.1349)
**Model E (D2_cm3_)**		
AUC	0.64	0.62 (0.57 – 0.67)
ICI	0.014	0.015 (0–0.035)
Calibration intercept	0.00	0.10 (−0.48 – 1.22)
Calibration slope	1.00	1.05 (0.72–1.67)
Brier score	0.1145	0.1159 (0.0979–0.1357)

Abbreviations: AUC = Area under the curve, ICI = Integrated Calibration Index.

The models show similar calibration measures with overlapping confidence intervals, with ICI’s of 0.020 (0 – 0.037), 0.007 (0 – 0.027) and 0.015 (0––0.035) and calibrations slopes of 1.01 (0.68 – 1.49), 1.01 (0.68–1.65) and 1.05 (0.72 – 1.67) for the EUD (n=0.1), D_0.1cm3_ and D_2cm3_-models, respectively. The AUC’s of 0.64 (0.58 – 0.69), 0.60 (0.55 – 0.65), 0.62 (0.57 – 0.67) and Brier scores are also similar.

### Sensitivity analysis

In the sensitivity analysis, models were fitted with various dose parameters and α/β’s. When an α/β of 2 Gy was applied, fractionation schedule was eliminated in models based on D_0.1cm3_, D_2cm3_, V147.1 (BED equivalent of physical V75 in CF) and EUDs with n=0.05, n=0.10, n=0.15, and n=0.20. When an α/β of 3 Gy was applied, fractionation schedule was only eliminated in the models based on D_0.1cm3_, D_2cm3_ and EUDs with n=0.05 and n=0.10. Since the performances of these models were similar, no dose parameter can be deemed the best. Section 3 of Supplementary Materials contains more details.

## Discussion

We developed NTCP models for grade ≥2 LRB after conventional or hypofractionated EBRT for prostate cancer. Our trial data set with different fractionation schedules offered the unique possibility to generate unbiased BED-based models. Models based on EUD(n=0.1), D_0.1cm3_ and D_2cm3_ were found to describe the dose–response for both fractionation schedules. Model performance, in terms of calibration and discrimination, was similar between these models.

The predictive value of EUD and previous abdominal surgery are in line with literature on rectal bleeding after conventional EBRT [8], [9], [10], [11], [12], [13], [14], [15], [16], [17], [18], [19], [20], [21]. D_0.1cm3_ and D_2cm3_ were found to be predictive for late rectal toxicity and bleeding after EBRT and BT for cervical cancer [22], [23]. Since rectal bleeding is considered to behave serially [1], their predictive roles in the D_xcc_-models are also as expected. In contrary to LRB after radiotherapy for gynecological cancer, it is uncommon to fit LRB after radiotherapy for prostate cancer using dose parameters describing the minimal dose within a fixed volume receiving the highest dose levels. The achieved models show a performance similar to the EUD(n=0.1) model.

Currently, an α/β ratio of 3 Gy for late normal tissue is generally assumed [33], [34], [35] and was recommended in the QUANTEC paper [42]. Brand et al (2021) used CHHIP trial data of over 2000 patients randomized to CF or HF to estimate the α/β ratio and the LKB parameters n, m, and TD50 for several late toxicity endpoints, including LRB [36]. They reported for Grade ≥2 LRB the optimal fit for an α/β ratio of 1.7 Gy (95% CI: 0.3–3.0 Gy). This model did however not significantly outperform a model with a fixed α/β ratio of 3 Gy. In the sensitivity analysis of the current study, fractionation schedule was eliminated by the backward scheme in four models when assuming α/β = 3 Gy and in seven models when α/β = 2 Gy was assumed (Section 3 of Supplementary Materials). Therefore, the assumption of α/β = 3 Gy in the main analysis appears to be appropriate but the true value might be closer to the 1.7 Gy of Brand et al. found, and our findings support their suggestion to use a late rectal α/β ratio of no more than 3 Gy [36]. During the design of the HYPRO trial, an α/β of 4 to 6 Gy was assumed [25]. In the initial report of the HYPRO study on late toxicity, late rectal bleeding was scored based on whether patients needed activated clotting time and no significant difference was found between arms [25]. In a more recent report that scored LRB using patient questionnaires, significantly more bleeders were found in the HF arm [27]. The HF prescription dose in BED is only larger than CF for an α/β of 4.7 Gy or smaller.

The EUD models in the current study were fit with n = 0.1 and n = 0.2. This choice was made based on the range of 0.06 and 0.24 reported in Fiorino’s review [1] and Cicchetti’s validation study [38]. This range also contains the best fit values for LRB models (0.06, 0.23,0.24 [8], 0.13 [9]) that are reported in the QUANTEC study [42]. It should be noted that these literature values are based on physical dose distributions. By converting the dose distributions to BED, voxel doses change relative to each other, which impacts optimal n. We found that an n of 0.2 (assuming α/β = 3 Gy) was suboptimal in describing dose–response relationship since fractionation schedule remained a significant factor, and a lower n of 0.1 gave a superior fit (Tables 1). However, when an α/β of 2 Gy was applied, fractionation schedule was eliminated for n values from 0.05 to 0.2 (Table S3).

Cicchetti et al. investigated the transportability and generalizability of EUD-based NTCP models for LRB by testing published models on their large pooled dataset of three previous trials [38]. They used patient-reported rectal bleeding for the Grade ≥2 LRB endpoint, which deviates from our study, where Grade ≥2 LRB is also scored based on the case report form. Assuming α/β = 3 Gy, they concluded that published models with n in the range of 0.18–0.24 showed superior fits with their data, suggesting that both medium and high-dose areas are important. This is somewhat in contradiction with our results, suggesting that high-dose volumes are most important, as observed with the EUD (n=0.1) and BED_xcm3_ models (Table 1). This might be due to the fact that we also scored Grade ≥2 LRB in case treatment was indicated.

With the optimal α/β ratio of 1.7 Gy, Brand et al. estimated parameter n = 0.16 in their study [36]. For α/β ratios fixed to 3 and 4.8 Gy, they estimated n = 0.19 and n = 0.24, respectively. For a scenario without fraction size adjustment their estimates were n = 0.13 for CF and n = 0.11 for HF. Our finding of a suboptimal n = 0.2 seems to contradict with their estimated optimal n of 0.19 with a fixed α/β ratio of 3 Gy. However, the confidence interval of their n estimation was quite large (0.03–0.36) and contained 0.1 as well.

Of note is that in the CHHIP trial, pelvic surgery appeared not significant, and cardiovascular disease / use of anticoagulants was not tested in their study [35]. The impact of the risk factors Cardiovascular disease and Abdominal surgery were confirmed in Cicchetti’s validation study [38].

Dennstädt et al published a systematic literature review on reported LKB parameters for various toxicity endpoints, including a large number of studies on LRB [43]. They conclude that LKB modeling (using EUD for a defined organ at risk to model an endpoint) is not always suitable to describe dose-reponse relationships, but seems to be a fairly accurate model for the LRB endpoint. And in general, such simplified modeling is clinically useful to assess the odds of a safe treatment.

The models in the current study were developed by combining CF and HF data. Wilkins et al. previously combined CF and HF data of the CHHiP trial in accordance with the linear quadratic model to derive anorectal dose constraints for various endpoints [44]. For LRB they found significant OR’s for dose cut-offs from 20 to 70 Gy in a univariate analysis. The purpose in the current study was not to derive constraints but to quantify dose–response. For that purpose the D_xcm3_ and EUD parameters were found to be more suitable than dose cut-off parameter V111.9 since fractionation schedule remained a factor in that model (Table 1). Cicchetti et al also concluded that EUD fits are superior to fits based on a single dose-volume cutoff value for describing the dose–response for LRB [38].

Limitations of our model development include the limited statistical power with a sample size of 656 patients and 89 events, and the lack of information on cardiovascular history / use of anticoagulants. A strong point of our dataset is its prospective nature and the availability of both conventional and hypofractionated data within the framework of the same randomized trial, such that the data collection was identical for both subgroups. We are planning to externally validate our obtained NTCP model in a future independent dataset for which data collection is currently ongoing (prospective cohort study) in patients treated in our clinic with 60 Gy in 20 fractions or 42.7 Gy in 7 fractions [45].

In conclusion, we demonstrated that, assuming an α/β of 3, the EUD model with n = 0.1 and the BED_xcm3_ models described the data well, whereas EUD models with n >=0.15 or models based on a high dose-volume cutoff, failed. Assuming an α/β ratio of 2 Gy, however, EUD models with n=0.15–0.20, or models based on high dose-volume cutoffs resulted in good fits as well. To establish the true combination of n and α/β value, further analyses of larger datasets with more variation in fractionation schedules are required.

## CRediT authorship contribution statement

**Christian A.M. Jongen:** Formal analysis, Methodology, Visualization, Writing – original draft. **Ben J.M. Heijmen:** Conceptualization, Investigation, Methodology, Supervision, Project administration, Writing – review & editing. **Wilco Schillemans:** Data curation, Writing – review & editing. **Andras Zolnay:** Data curation, Writing – review & editing, Software. **Marnix G. Witte:** Conceptualization, Investigation, Methodology, Writing – review & editing. **Floris J. Pos:** Investigation, Methodology, Writing – review & editing. **Ben Vanneste:** Conceptualization, Methodology, Writing – review & editing. **Ludwig J. Dubois:** Writing – review & editing. **David van Klaveren:** Methodology, Writing – review & editing. **Luca Incrocci:** Conceptualization, Investigation, Supervision, Writing – review & editing. **Wilma D. Heemsbergen:** Conceptualization, Data curation, Funding acquisition, Investigation, Methodology, Project administration, Supervision, Writing – original draft, Writing – review & editing.

## Declaration of competing interest

The authors declare the following financial interests/personal relationships which may be considered as potential competing interests: This research was funded by the Dutch Cancer Society (KWF, project nr 14259).

## Data Availability

The data that support the findings of this study are available from the corresponding author [WH] on request.
