# Supplementary material for: Normal tissue complication probability modeling for late rectal bleeding after conventional or hypofractionated radiotherapy for prostate cancer
Source: Clin Transl Radiat Oncol. 2024 Nov 10;50:100886. doi: 10.1016/j.ctro.2024.100886 (PMC11701999; doi:10.1016/j.ctro.2024.100886)
Supplement: Supplementary Data 1 [file mmc1.docx]

**Supplementary Material**

Section 1. Biological Effective Dose and patient characteristics.

**
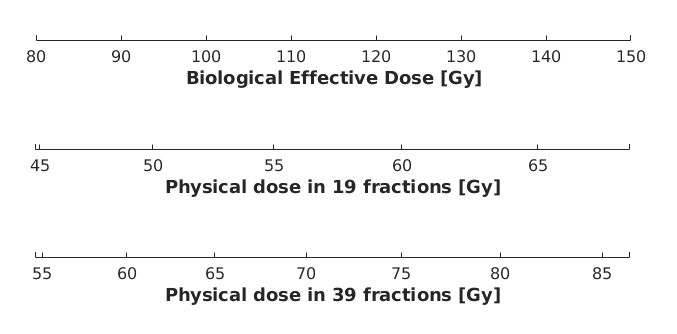
**

**Figure S1.** Biological Effective Dose and corresponding physical dose in 19 and 39 fractions for α/β = 3 Gy.

**Table S1.** Patient characteristics at the start of treatment.

| **Variable** | **N (%) of total group (N=656)** |
| --- | --- |
| Age (mean, standard deviation) in years | 70.0 (6.2) |
| TURP | 63 (10) |
| Previous abdominal surgery | 173 (26) |
| Diabetes | 89 (14) |
| Adjuvant hormonal therapy | 430 (66) |
| Conventional fractionation 39x2 Gy | 331 (50) |
| Hypofractionation 19x3.4 Gy | 325 (50) |

Abbreviation: TURP = transurethral resection of prostate

Section 2. Models expressions

The obtained models for grade ≥2 LRB were fitted to express NTCP as follows:

$$NTCP =\frac{1}{1+e^{-S}}$$

With HYPOTREAT = 0 or 1 for CF or HF, respectively, and ABD SURG = 0 or 1 for previous abdominal surgery or no previous abdominal surgery, respectively.

| **Model** | **NTCP** |
| --- | --- |
| **A** | S = -2.95 + 5.04 ⋅ V111.9 + 0.51 ⋅ HYPOTREAT + 0.57 ⋅ ABD SURG |
| **B** | B: S = -8.94 + 0.068 ⋅ EUD(n=0.1) + 0.59 ⋅ ABD SURG |
| **C** | S = -6.02 + 0.043 ⋅ EUD(n=0.2) + 0.46 ⋅ HYPOTREAT + 0.59 ⋅ ABD SURG |
| **D** | S = -8.01 + 0.044 ⋅ D0.1cm3 + 0.54 ⋅ ABD SURG |
| **E** | S = -8.24 + 0.048 ⋅ D2cm3 + 0.53 ⋅ ABD SURG |

**Table S2.** Expressions of the final models.

**
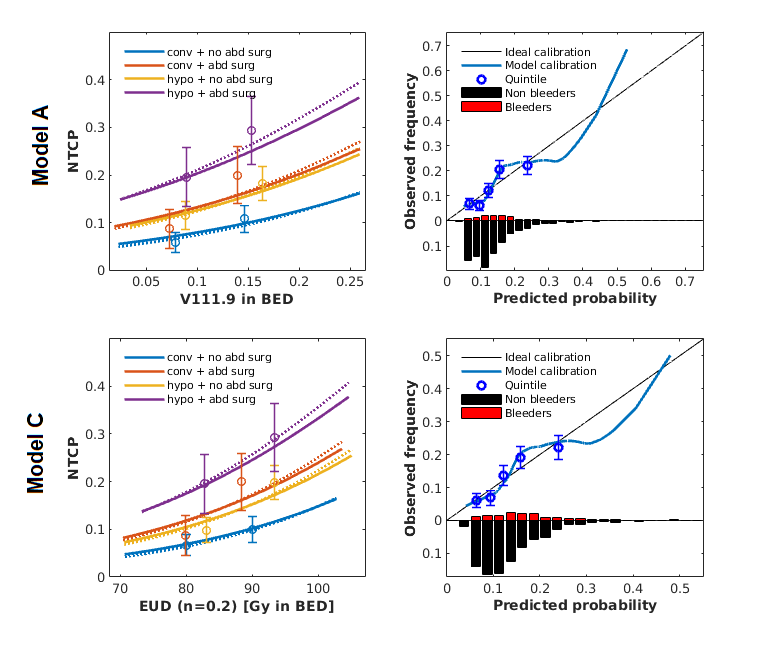
**

**Figure S2.** The models based on V111.9 and EUD (n=0.2) (A & C). The dashed line shows the model before model coefficients were shrunk. The calibration plots show the apparent calibration based on quintiles and loess curve for the unshrunk models. The error bars show standard deviations. The histogram shows the distribution of predicted probabilities for bleeders and non-bleeders. Probabilities of outliers were not included in the model plot, but were in the calibration plots.

Section 3. Sensitivity analysis

Based on literature, we assumed α/β = 3 Gy, and (for the fitted EUD models) a n value of 0.1 and 0.2, and we evaluated the dose volume parameter V111.9 as a BED equivalent of the physical V70. To study the impact of these choices on the final models and their performance, a sensitivity analysis was performed.

In total 60 extra models were fitted, each with one dose parameter, previous abdominal surgery and fractionation schedule as candidate predictors. Tested alternative α/β values were in the range of 2-6 Gy which correspond with alternative values reported in literature [33,34,35,36]. Models were fitted with the same five dose-volume parameters as were selected for the main analysis and with the same approach, but calculated for α/β = 2, 4, 5 and 6 Gy, resulting in 20 new models. Another 40 model were fitted with alternative dose parameters that were not selected in the main analysis: EUD’s in BED with n = 0.05, 0.15 and 0.25 and the BED equivalents of physical V50, V55, V60, V65 and V75 calculated for α/β’s = 2, 3, 4, 5 and 6 Gy.

A final sensitivity analysis concerned a separate fit of the HF and SF data set, in order to assess whether the slopes of the separate fits were comparable with the slopes of the joined fits.

Tables S3, S4, and S5 show the measures of performance and whether fractionation schedule was a predictor in the final models. Fractionation schedule remained final predictor in all models in which the dose parameter was converted to BED with α/β-ratios of 4, 5 or 6 Gy, suggesting that applying these α/β ratios is inappropriate to account for fractionation effects. Fractionation schedule was eliminated for the EUD (n=0.05), EUD (n=0.10), D_0.1cm3_ and D_2cm3_-based models when α/β = 2 or 3 Gy was assumed. In the models based on EUD (n=0.15), EUD (n=0.20) and the BED equivalent of V75, however, fractionation schedule was only eliminated when α/β = 2 Gy was assumed. Although fractionation schedule was eliminated in more models when α/β = 2 Gy was assumed, the performance was not better than for the models where α/β = 3 Gy was assumed.

To further corroborate the validity of the EUD (n=0.1), D_0.1cm3_ and D_2cm3_-models for both fractionation schedules, models with the same candidate predictors were fitted to the CF and HF data separately (Table S6). Only for the EUD (n=0.1)-model this resulted in two models in which the dose parameter was a significant predictor (p-value < 0.05) in the final model. The OR’s of 1.07 and 1.05 for EUD(n=0.1) that were found fitting CF and HF data separately are similar to the 1.07 that was found when their combined data was fitted in the EUD (n=0.1)-model (table 1).

**Table S3**. Whether fractionation schedule (HT) is a predictor in the final model (yes/no) and corrected performance metrics for EUD-based models in the sensitivity analysis. Models in which fractionation schedule was not a final predictor are highlighted in blue.

| 6 | HT: Yes  AUC:0.63 (0.58 – 0.68)  ICI: 0.014 (0 – 0.034)  Cal In: 0.02 (-0.56 – 1.07)  Cal Sl: 1.01 (0.68 – 1.59)  Br Sc: 0.1154 (0.0977 – 0.1344) | HT: Yes  AUC: 0.65 (0.59 – 0.70)  ICI: 0.019 (0.001 – 0.037)  Cal In: 0.04(-0.48 – 0.97)  Cal Sl.: 1.02 (0.72 – 1.54)  Br Sc: 0.1147 (0.0958 – 0.1348) | HT: Yes  AUC: 0.64 (0.59 – 0.70)  ICI: 0.018 (0 – 0.036)  Cal In: 0.02 (-0.49 – 0.93)  Cal Sl.: 1.01 (0.72 – 1.52)  Br Sc: 0.1148 (0.0980 – 0.1334) | HT: Yes  AUC: 0.64 (0.59 – 0.70)  ICI: 0.016 (0 – 0.033)  Cal In: 0.05 (-0.48 – 1.03)  Cal Sl: 1.03 (0.72 – 1.57)  Br Sc: 0.1152 (0.0978 – 0.1338) | HT: Yes  AUC: 0.64 (0.58 – 0.69)  ICI: 0.015 (0 – 0.034)  Cal In: 0.11 (-0.43 – 1.25)  Cal Sl: 1.06 (0.75 – 1.70)  Br Sc: 0.1145 (0.0972 – 0.1328) |
| --- | --- | --- | --- | --- | --- |
| 5 | HT: Yes  AUC:0.63 (0.58 – 0.68)  ICI: 0.014 (0 – 0.030)  Cal In: 0.00 (-0.53 – 0.92)  Cal Sl: 1.00 (0.69 – 1.53)  Br Sc: 0.1154 (0.0960 – 0.1351) | HT: Yes  AUC: 0.64 (0.59 – 0.69)  ICI: 0.019 (0.002 – 0.036)  Cal In: 0.04 (-0.46 – 0.99)  Cal Sl.: 1.02 (0.74 – 1.57)  Br Sc: 0.1146 (0.0961 – 0.1333) | HT: Yes  AUC: 0.64 (0.59 – 0.70)  ICI: 0.019 (0.000 – 0.038)  Cal In: 0.03 (-0.51 – 0.92)  Cal Sl.: 1.02 (0.71 – 1.52)  Br Sc: 0.1147 (0.0964 – 0.1336) | HT: Yes  AUC: 0.64 (0.59 – 0.69)  ICI: 0.016 (0 – 0.034)  Cal In: 0.03 (-0.51 – 0.89)  Cal Sl: 1.01 (0.70 – 1.49)  Br Sc: 0.1144 (0.0956 – 0.1331) | HT: Yes  AUC: 0.64 (0.59 – 0.69)  ICI: 0.015 (0 – 0.033)  Cal In: 0.06 (-0.47 – 0.98)  Cal Sl: 1.03 (0.73 – 1.55)  Br Sc: 0.1146 (0.0969 – 0.1331) |
| 4 | HT: Yes  AUC:0.63 (0.57 – 0.68)  ICI: 0.015 (0 – 0.035)  Cal In: 0.06 (-0.48 – 1.00)  Cal Sl: 1.03 (0.72 – 1.56)  Br Sc: 0.1152 (0.0978 – 0.1331) | HT: Yes  AUC: 0.64 (0.59 – 0.70)  ICI: 0.019 (0 – 0.036)  Cal In: -0.01 (-0.53 – 0.92)  Cal Sl.: 1.00 (0.69 – 1.51)  Br Sc: 0.1148 (0.0968 – 0.1332) | HT: Yes  AUC: 0.64 (0.58 – 0.70)  ICI: 0.019 (0.001 – 0.036)  Cal In: 0.01 (-0.52 – 0.95)  Cal Sl.: 1.00 (0.70 – 1.54)  Br Sc: 0.1149 (0.0952 – 0.1340) | HT: Yes  AUC: 0.64 (0.59 – 0.70)  ICI: 0.017 (0 – 0.035)  Cal In: 0.04 (-0.50 – 0.92)  Cal Sl: 1.02 (0.71 – 1.52)  Br Sc: 0.1148 (0.0968 – 0.1340) | HT: Yes  AUC: 0.64 (0.59 – 0.69)  ICI: 0.015 (0 – 0.032)  Cal In: 0.02 (-0.51 – 0.92)  Cal Sl: 1.01 (0.71 – 1.52)  Br Sc: 0.1153 (0.0958 – 0.1349) |
| 3 | HT: No  AUC:0.63 (0.57 – 0.68)  ICI: 0.013 (0 – 0.030)  Cal In: 0.00 (-0.58 – 1.07)  Cal Sl: 1.00 (0.67 – 1.59)  Br Sc: 0.1153 (0.0966 – 0.1350) | HT: No  AUC: 0.64 (0.58 – 0.69)  ICI: 0.019 (0.000 – 0.037)  Cal In: 0.02 (-0.54 – 0.88)  Cal Sl.: 1.01 (0.68 – 1.49)  Br Sc: 0.1151 (0.0964 – 0.1325) | HT: Yes  AUC: 0.64 (0.59 – 0.70)  ICI: 0.019 (0.000 – 0.037)  Cal In: 0.01 (-0.51 – 0.89)  Cal Sl.: 1.01 (0.71 – 1.51)  Br Sc: 0.1150 (0.0971 – 0.1343) | HT: Yes  AUC: 0.64 (0.58 – 0.69)  ICI: 0.017 (0 – 0.034)  Cal In: 0.03 (-0.48 – 0.95)  Cal Sl: 1.02 (0.72 – 1.52)  Br Sc: 0.1144 (0.0969 – 0.1331) | HT: Yes  AUC: 0.64 (0.59 – 0.69)  ICI: 0.014 (0 – 0.032)  Cal In: -0.02 (-0.52 – 0.85)  Cal Sl: 0.99 (0.70 – 1.47)  Br Sc: 0.1145 (0.0958 – 0.1326) |
| 2 | HT: No  AUC:0.63 (0.58 – 0.69)  ICI: 0.014 (0 – 0.031)  Cal In: 0.01 (-0.55 – 1.16)  Cal Sl: 1.00 (0.68 – 1.65)  Br Sc: 0.1156 (0.0970 – 0.1352) | HT: No  AUC: 0.64 (0.59 – 0.70)  ICI: 0.019 (0 – 0.037)  Cal In: 0.01 (-0.54 – 1.01)  Cal Sl.: 1.01 (0.68 – 1.56)  Br Sc: 0.1146 (0.0962 – 0.1327) | HT: No  AUC: 0.65 (0.59 – 0.70)  ICI: 0.021 (0 – 0.040)  Cal In: 0.00 (-0.52 – 0.89)  Cal Sl.: 1.00 (0.70 – 1.50)  Br Sc: 0.1146 (0.0960 – 0.1335) | HT: No  AUC: 0.64 (0.59 – 0.70)  ICI: 0.017 (0 – 0.033)  Cal In: 0.01 (-0.52 – 1.05)  Cal Sl: 1.00 (0.70 – 1.59)  Br Sc: 0.1146 (0.0964 – 0.1335) | HT: Yes  AUC: 0.64 (0.59 – 0.70)  ICI: 0.016 (0 – 0.032)  Cal In: 0.02 (-0.50 – 1.10)  Cal Sl: 1.01 (0.70 – 1.61)  Br Sc: 0.1156 (0.0976 – 0.1340) |
| α/β (Gy) → Dose parameter↓ | EUD (n=0.05) | EUD (n=0.10) | EUD (n=0.15) | EUD (n=0.20) | EUD (n=0.25) |

Abbreviations: EUD = Equivalent Uniform Dose, HT = HypoTreat, AUC = Area under the curve, ICI = Integrated Calibration Index, Cal In: calibration intercept, Cal Sl: calibration slope, Br Sc: Brier score

**Table S4.** Whether treatment (HT) is a predictor in the final model (yes/no) and corrected performance metrics for V_D_-based models in the sensitivity analysis. Models in which fractionation schedule was not a final predictor are highlighted in blue.

| 6 | V60.7 in BED  HT: Yes  AUC: 0.62 (0.57 – 0.67)  ICI: 0.013 (0 – 0.032)  Cal In: 0.00 (-0.55 – 0.93)  Cal Sl: 1.00 (0.69 – 1.53)  Br Sc: 0.1159 (0.0979 – 0.1354) | V67.9 in BED  HT: Yes  AUC: 0.63 (0.58 – 0.68)  ICI: 0.016 (0 – 0.034)  Cal In: 0.06 (-0.49 – 1.15)  Cal Sl: 1.03 (0.72 – 1.64)  Br Sc: 0.1157 (0.0977 – 0.1339) | V75.4 in BED  HT: Yes  AUC: 0.63 (0.58 – 0.68)  ICI: 0.019 (0 – 0.039)  Cal In: 0.05 (-0.49 – 1.08)  Cal Sl: 1.02 (0.72 – 1.62)  Br Sc: 0.1145 (0.0971 – 0.1345) | V83.1 in BED  HT: Yes  AUC: 0.64 (0.58 – 0.69)  ICI: 0.020 (0.001 – 0.038)  Cal In: -0.01 (-0.55 – 0.98)  Cal Sl: 1.00 (0.69 – 1.54)  Br Sc: 0.1151 (0.0968 – 0.1331) | V90.9 in BED  HT: Yes  AUC: 0.64 (0.58 – 0.69)  ICI: 0.021 (0.004 – 0.038)  Cal In: 0.02 (-0.51 – 0.93)  Cal Sl: 1.01 (0.71 – 1.53)  Br Sc: 0.1145 (0.0968 – 0.1331) | V99.0 in BED  HT: Yes  AUC: 0.63 (0.57 – 0.68)  ICI: 0.013 (0 – 0.032)  Cal In: 0.05 (-0.49 – 1.19)  Cal Sl: 1.03 (0.72 – 1.68)  Br Sc: 0.1153 (0.0974 – 0.1351) |
| --- | --- | --- | --- | --- | --- | --- |
| 5 | V62.8 in BED  HT: Yes  AUC: 0.62 (0.57 – 0.67)  ICI: 0.013 (0 – 0.032)  Cal In: 0.04 (-0.56 – 1.03)  Cal Sl: 1.02 (0.68 – 1.57)  Br Sc: 0.1159 (0.0985 – 0.1352) | V70.5 in BED  HT: Yes  AUC: 0.63 (0.58 – 0.68)  ICI: 0.016 (0 – 0.034)  Cal In: 0.03 (-0.54 – 1.02)  Cal Sl: 1.02 (0.68 – 1.57)  Br Sc: 0.1152 (0.0958 – 0.1339) | V78.5 in BED  HT: Yes  AUC: 0.63 (0.58 – 0.68)  ICI: 0.018 (0 – 0.037)  Cal In: 0.02 (-0.53 – 0.99)  Cal Sl: 1.01 (0.69 – 1.55)  Br Sc: 0.1153 (0.0980 – 0.1341) | V86.7 in BED  HT: Yes  AUC: 0.64 (0.59 – 0.69)  ICI: 0.021 (0.002 – 0.039)  Cal In: 0.03 (-0.50 – 1.07)  Cal Sl: 1.02 (0.71 – 1.59)  Br Sc: 0.1151 (0.0975 – 0.1344) | V95.1 in BED  HT: Yes  AUC: 0.64 (0.59 – 0.69)  ICI: 0.019 (0.001 – 0.037)  Cal In: -0.02 (-0.57 – 0.94)  Cal Sl: 0.99 (0.68 – 1.53)  Br Sc: 0.1147 (0.0971 – 0.1334) | V103.9 in BED  HT: Yes  AUC: 0.63 (0.57 – 0.68)  ICI: 0.017 (0 – 0.036)  Cal In: -0.01 (-0.55 – 0.98)  Cal Sl: 1.00 (0.69 – 1.55)  Br Sc: 0.1152 (0.0977 – 0.1336) |
| 4 | V66.0  HT: Yes  AUC: 0.62 (0.56 – 0.67)  ICI: 0.013 (0 – 0.032)  Cal In: 0.04 (-0.55 – 1.05)  Cal Sl: 1.02 (0.68 – 1.57)  Br Sc: 0.1157 (0.0990 – 0.1339) | V74.4 in BED  HT: Yes  AUC: 0.63 (0.57 – 0.67)  ICI: 0.016 (0 – 0.034)  Cal In: 0.07 (-0.49 – 1.01)  Cal Sl: 1.04 (0.72 – 1.57)  Br Sc: 0.1158 (0.0969 – 0.1346) | V83.1 in BED  HT: Yes  AUC: 0.63 (0.58 – 0.68)  ICI: 0.018 (0 – 0.037)  Cal In: 0.03 (-0.52 – 0.98)  Cal Sl: 1.02 (0.70 – 1.55)  Br Sc: 0.1160 (0.0983 – 0.1342) | V92.1 in BED  HT: Yes  AUC: 0.64 (0.58 – 0.69)  ICI: 0.021 (0.001 – 0.040)  Cal In: 0.07 (-0.49 – 1.16)  Cal Sl: 1.04 (0.72 – 1.66)  Br Sc: 0.1151 (0.0975 – 0.1329) | V101.4 in BED  HT: Yes  AUC: 0.64 (0.59 – 0.70)  ICI: 0.020 (0.001 – 0.038)  Cal In: 0.03 (-0.54 – 0.95)  Cal Sl: 1.01 (0.69 – 1.53)  Br Sc: 0.1146 (0.0962 – 0.1350) | V111.1 in BED  HT: Yes  AUC: 0.63 (0.58 – 0.69)  ICI: 0.019 (0.001 – 0.038)  Cal In: 0.03 (-0.49 – 0.98)  Cal Sl: 1.02 (0.72 – 1.55)  Br Sc: 0.1151 (0.0978 – 0.1331) |
| 3 | V71.4 in BED  HT: Yes  AUC: 0.62 (0.57 – 0.67)  ICI: 0.012 (0 – 0.032)  Cal In: 0.02 (-0.55 – 1.08)  Cal Sl: 1.01 (0.69 – 1.60)  Br Sc: 0.1155 (0.0974 – 0.1345) | V80.9 in BED  HT: Yes  AUC: 0.62 (0.57 – 0.67)  ICI: 0.014 (0 – 0.033)  Cal In: 0.02 (-0.53 – 0.98)  Cal Sl: 1.01 (0.69 – 1.55)  Br Sc: 0.1157 (0.0982 – 0.1355) | V90.8 in BED  HT: Yes  AUC: 0.63 (0.58 – 0.68)  ICI: 0.017 (0 – 0.035)  Cal In: 0.1 (-0.42 – 1.13)  Cal Sl: 1.06 (0.76 – 1.62)  Br Sc: 0.1154 (0.0972 – 0.1336) | V101.1 in BED  HT: Yes  AUC: 0.63 (0.58 – 0.68)  ICI: 0.019 (0.002 – 0.038)  Cal In: -0.03 (-0.58 – 0.95)  Cal Sl: 0.98 (0.67 – 1.53)  Br Sc: 0.1161 (0.0986 – 0.1347) | V111.9 in BED  HT: Yes  AUC: 0.64 (0.59 – 0.70)  ICI: 0.020 (0.002 – 0.038)  Cal In: 0.06 (-0.45 – 0.95)  Cal Sl: 1.03 (0.74 – 1.54)  Br Sc: 0.1153 (0.0967 – 0.1349) | V123.1 in BED  HT: Yes  AUC: 0.64 (0.58 – 0.69)  ICI: 0.018 (0.002 – 0.036)  Cal In: 0.03 (-0.51 – 1.13)  Cal Sl: 1.02 (0.71 – 1.61)  Br Sc: 0.1146 (0.0961 – 0.1329) |
| 2 | V82.1 in BED  HT: Yes  AUC: 0.62 (0.56 – 0.67)  ICI: 0.013 (0 – 0.034)  Cal In: 0.01 (-0.59 – 1.23)  Cal Sl: 1.01 (0.67 – 1.67)  Br Sc: 0.1163 (0.0986 – 0.1350) | V93.8 in BED  HT: Yes  AUC: 0.62 (0.57 – 0.67)  ICI: 0.014 (0 – 0.032)  Cal In: 0.02 (-0.51 – 0.96)  Cal Sl: 1.01 (0.71 – 1.54)  Br Sc: 0.1154 (0.0973 – 0.1338) | V106.2 in BED  HT: Yes  AUC: 0.63 (0.57 – 0.68)  ICI: 0.014 (0 – 0.032)  Cal In: 0.01 (-0.55 – 1.08)  Cal Sl: 1.01 (0.69 – 1.60)  Br Sc: 0.1156 (0.0977 – 0.1353) | V119.2 in BED  HT: Yes  AUC: 0.63 (0.58 – 0.68)  ICI: 0.018 (0 – 0.036)  Cal In: 0.05 (-0.48 – 1.02)  Cal Sl: 1.02 (0.72 – 1.56)  Br Sc: 0.1157 (0.0987 – 0.1346) | V132.8 in BED  HT: Yes  AUC: 0.64 (0.58 – 0.69)  ICI: 0.020 (0.002 – 0.037)  Cal In: 0.00 (-0.51 – 0.92)  Cal Sl: 1.00 (0.71 – 1.52)  Br Sc: 0.1154 (0.0964 – 0.1345) | V147.1 in BED  HT: No  AUC: 0.64 (0.58 – 0.69)  ICI: 0.015 (0 – 0.033)  Cal In: 0.00 (-0.54 – 0.94)  Cal Sl: 1,00 (0.69 – 1.52)  Br Sc: 0.1149 (0.0969 – 0.1333) |
| α/β (Gy)→ Physical Dose parameter in CF (Gy)↓ | V50 | V55 | V60 | V65 | V70 | V75 |

Abbreviations: HT = HypoTreat, AUC = Area under the curve, ICI = Integrated Calibration Index, Cal In: calibration intercept, Cal Sl: calibration slope, Br Sc: Brier score

**Table S5.** Whether fractionation schedule (HT) is a predictor in the final model (yes/no) and corrected performance metrics for Dxcm3-based models in the sensitivity analysis. Models in which fractionation schedule was not a final predictor are highlighted in blue.

| Dose parameter → α/β (Gy)↓ | D0.1cm3 | D2cm3 |
| --- | --- | --- |
| 2 | HT: No  AUC: 0.61 (0.56 – 0.66)  ICI: 0.010 (0 – 0.029)  Cal In: -0.01 (-0.64 – 1.22)  Cal. Sl.: 0.99 (0.64 – 1.67)  Br. Sc: 0.1157 (0.0981 – 0.1343) | HT: No  AUC: 0.63 (0.57 – 0.68)  ICI: 0.014 (0 – 0.033)  Cal In: 0.06 (-0.54 – 1.16)  Cal. Sl.: 1.03 (0.70 – 1.64)  Br. Sc: 0.156 (0.0981 – 0.1351) |
| 3 | HT: No  AUC: 0.60 (0.55 – 0.65)  ICI: 0.007 (0 – 0.027)  Cal In: 0.01 (-0.56 – 1.19)  Cal. Sl.: 1.01 (0.68 – 1.65)  Br. Sc: : 0.1160 (0.0983 – 0.1349) | HT: No  AUC: 0.62 (0.57 – 0.67)  ICI: 0.015 (0 – 0.035)  Cal In: 0.10 (-0.48 – 1.22)  Cal. Sl.: 1.05 (0.72 – 1.67)  Br. Sc: : 0.1159 (0.0979 – 0.1357) |
| 4 | HT: Yes  AUC: 0.61 (0.56 – 0.66)  ICI: 0.012 (0 – 0.032)  Cal In: 0.03 (-0.52 – 1.19)  Cal. Sl.: 1.02 (0.71 – 1.66)  Br. Sc: 0.1161 (0.0983 – 0.1351) | HT: Yes  AUC: 0.62 (0.57 – 0.67)  ICI: 0.014 (0 – 0.033)  Cal In: 0.03 (-0.51 – 1.18)  Cal. Sl.: 1.02 (0.71 – 1.66)  Br. Sc: 0.1158 (0.0990 – 0.1350) |
| 5 | HT: Yes  AUC: 0.59 (0.54 – 0.64)  ICI: 0.003 (0 – 0.022)  Cal In: 0.00 (-0.58 – 1.00)  Cal. Sl.: 1.00 (0.67 – 1.56)  Br. Sc: 0.1162 (0.0971 – 0.1349) | HT: Yes  AUC: 0.62 (0.57 – 0.67)  ICI: 0.016 (0 – 0.034)  Cal In: 0.08 (-0.46 – 1.02)  Cal. Sl.: 1.04 (0.74 – 1.55)  Br. Sc: 0.1154 (0.0968 – 0.1342) |
| 6 | HT: Yes  AUC: 0.61 (0.55 – 0.66)  ICI: 0.011 (0 – 0.032)  Cal In: -0.02 (-0.59 – 1.19)  Cal. Sl.: 0.99 (0.66 – 1.66)  Br. Sc: 0.1158 (0.0978 – 0.1355) | HT: Yes  AUC: 0.62 (0.56 – 0.67)  ICI: 0.015 (0 – 0.033)  Cal In: 0.00 (-0.57 – 1.16)  Cal. Sl.: 1.00 (0.67 – 1.63)  Br. Sc: 0.1156 (0.0983 – 0.1351) |

Abbreviations: HT = HypoTreat, AUC = Area under the curve, ICI = Integrated Calibration Index, Cal In: calibration intercept, Cal Sl: calibration slope, Br Sc: Brier score

**Table S6**. Models by fitting CF and HF data separately with the same candidate predictors as the EUD (n=0.1), D_0.1cm3_ and D_2cm3_-models (B,D and E).

| Fitted models before shrinkage | | | | Final models after shrinkage |
| --- | --- | --- | --- | --- |
| Model | **Predictors** | **OR (95% CI)** | **p-value** | **OR** |
| B - CF only | EUD(n=0.1)  ABD SURG | 1.08 (1.00 – 1.16)  1.93 (0.91– 4.10) | 0.040  0.088 | 1.07 1.73 |
| B - HF only | EUD(n=0.1)  ABD SUR7 | 1.05 (1.00 – 1.11)  1.87 (1.00 – 3.49) | 0.032  0.048 | 1.05 1.69 |
| D - CF only | D_0.1cm3_  ABD SURG | 1.73 (0.98 – 1.14)  1.86 (0.88 – 3.96) | 0.157  0.102 | 1.52  1.61 |
| D - HF only | ABD SURG | 1.85 (1.00 – 3.44) | 0.049 | 1.65 |
| E - CF only | D_2cm3_  ABD SURG | 1.06 (0.99 – 1.14)  1.84 (0.87 – 3.91) | 0.115  0.109 | 1.05  1.61 |
| E - HF only | ABD SURG | 1.85 (1.00 – 3.44) | 0.049 | 1.62 |
